# Disruption of the glomerular basement membrane associated with nutcracker syndrome and double inferior vena cava in Noonan syndrome: a case report

**DOI:** 10.1186/s12882-022-02671-4

**Published:** 2022-02-12

**Authors:** Ayumi Omori, Kan Katayama, Ryosuke Saiki, Satoru Masui, Kei Suzuki, Yoshinori Kanii, Kayo Tsujimoto, Shiro Nakamori, Tairo Kurita, Tomohiro Murata, Takahiro Inoue, Kaoru Dohi

**Affiliations:** 1 Department of Cardiology and Nephrology, Mie University Graduate School of Medicine, 2-174 Edobashi, Tsu, Mie 514-8507 Japan; 2 Department of Nephro-Urologic Surgery and Andrology, Mie University Graduate School of Medicine, Tsu, Japan; 3 Department of Hematology and Oncology, Mie University Graduate School of Medicine, Tsu, Japan; 4 Department of Radiology, Mie University Hospital, Tsu, Japan

**Keywords:** Cystoscopy, *COL4A3*, Doppler ultrasonography, Hematuria, Proteinuria, von Willebrand

## Abstract

**Background:**

Nutcracker syndrome (NCS) is characterized by compression of the left renal vein (LRV) between the aorta and the superior mesenteric artery. While rare, NCS was reported to be accompanied by double inferior vena cava (IVC). We herein report a case of Noonan syndrome (NS) with double IVC who presented with macrohematuria and proteinuria.

**Case presentation:**

The patient was a 23-year-old man, who had been diagnosed with NS due to *RIT1* mutation, after showing foamy macrohematuria 3 weeks previously. A physical examination revealed low-set ears and a webbed neck. A urinalysis showed hematuria and proteinuria, and urinary sediments showed more than 100 isomorphic red blood cells per high-power field. His proteinuria and albuminuria concentrations were 7.1 and 4.5 g/g⋅Cr, respectively. Three-dimensional contrast-enhanced computed tomography (CT) showed double IVC and narrowing of the LRV after interflow of the left IVC. The aortomesenteric angle on a sagittal reconstruction of the CT image was 14.7°. Cystoscopy revealed a flow of macrohematuria from the left ureteral opening. On Doppler ultrasonography, there was scant evidence to raise the suspicion of the nutcracker phenomenon. Since severe albuminuria continued, a left kidney biopsy was performed. Light microscopy showed red blood cells in Bowman’s space and the tubular lumen. Electron microscopy revealed disruption of the glomerular basement membrane (GBM). Vulnerability of the GBM was suspected and a genetic analysis revealed a heterozygous mutation at c.4793 T > G (p.L1598R) in the *COL4A3* gene. Screening for coagulation disorders revealed the factor VIII and von Willebrand factor (vWF) values were low, at 47.6 and 23%, respectively. A multimer analysis of vWF showed a normal multimer pattern and he was diagnosed with von Willebrand disease type 1. As the bleeding tendency was mild, replacement of factor VIII was not performed. His macrohematuria and proteinuria improved gradually without treatment, and his urinalysis results have been normal for more than 6 months.

**Conclusions:**

The present case showed macrohematuria and proteinuria due to NCS in NS with double IVC and von Willebrand disease type 1. The macrohematuria and proteinuria originated from glomerular hemorrhage because of vulnerability of the GBM due to *COL4A3* mutation.

**Supplementary Information:**

The online version contains supplementary material available at 10.1186/s12882-022-02671-4.

## Background

Nutcracker syndrome (NCS) is characterized by compression of the left renal vein (LRV) between the aorta and the superior mesenteric artery [1]. An accurate diagnosis of NCS is sometimes difficult to make since a distended LRV can be observed as a normal anatomic variant [2]. Kim et al. proposed four different diagnostic criteria for NCS: the beak sign, the beak angle, the ratio of the LRV diameters, and angle between the superior mesenteric artery and the aorta [3]. Regarding the aortomesenteric angle on a sagittal reconstruction of computed tomography (CT), the angle < 41° was proposed for the definitive diagnosis [3].

The incidence of double inferior vena cava (IVC) is approximately 0.3–2.8% [4]. The left IVC usually terminates at the LRV, which passes anterior to the aorta to join the right IVC [5]. While rare, NCS was reported to be accompanied by left IVC or double IVC [6–9].

Noonan syndrome (NS) is an autosomal dominant disease caused by mutations in genes related to RAS/mitogen-activated protein kinase (MAPK) signaling pathway, such as *PTPN11, SOS1, RAF1, KRAS, NRAS, BRAF,* and *RIT1* [10]. Approximately 79% (153/194) of patients with NS were reported to have clotting factor deficiencies, von Willebrand disease, or platelet-related disorders [11].

## Case presentation

**Fig. 1:**
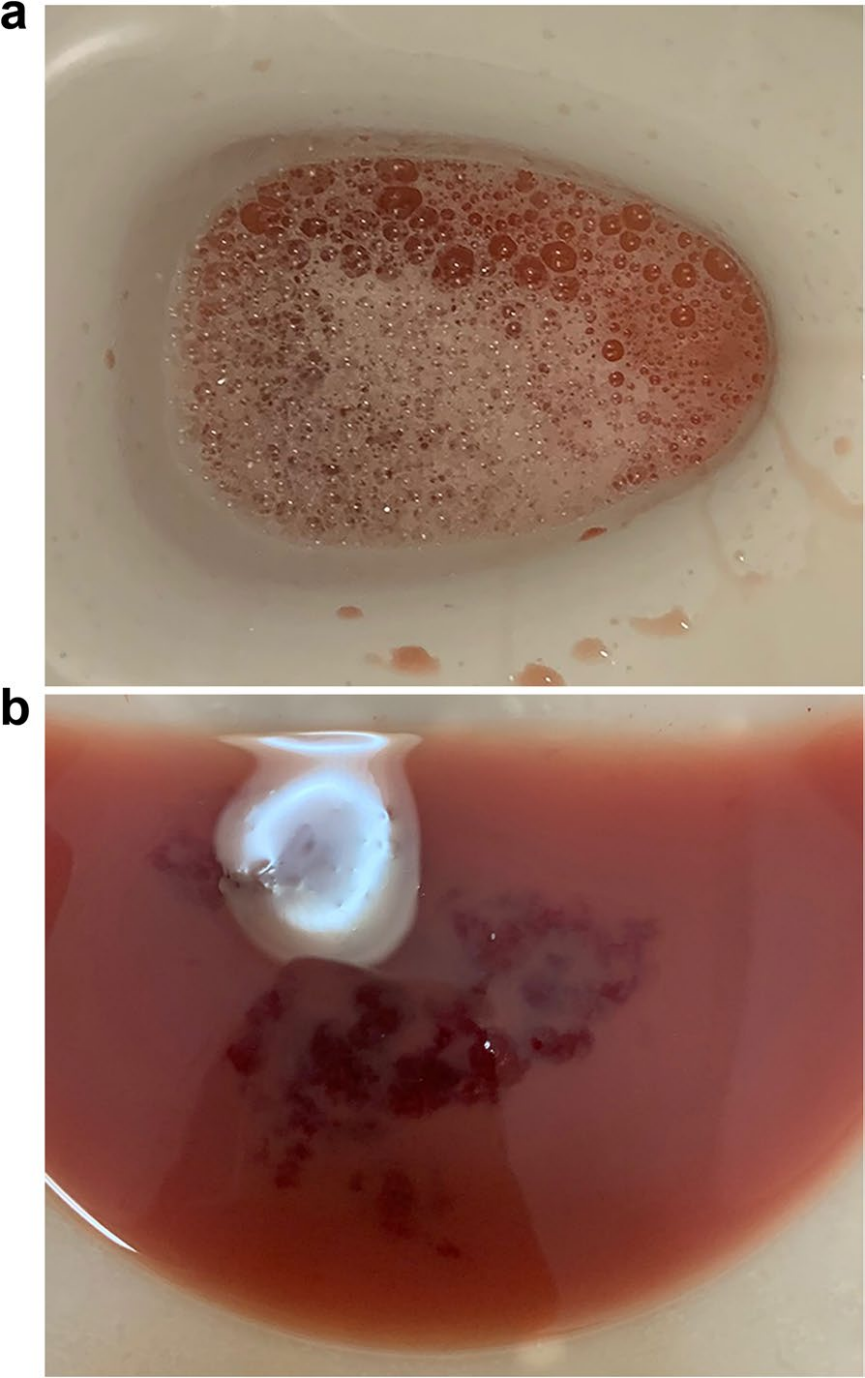
(**a**) Foamy macrohematuria. (**b**) A chunk of blood was observed in the macrohematuria

A 23-year-old man, who had been diagnosed with NS due to a heterozygous variant at c.335G > C (p.G112A) in the *RIT1* gene, developed foamy macrohematuria 3 weeks previously after driving for 6 h (Fig. 1a, b). He had no history of either left flank trauma or pain before the onset of the macrohematuria. A physical examination revealed low-set ears and a webbed neck, without mental retardation. Since he had received growth hormone replacement therapy, he did not have short stature; his height was 164.3 cm, and his body weight was 56.1 kg (this had not changed recently). Mild pulmonary valve stenosis was followed up without any intervention.

His body temperature, pulse rate, and blood pressure on arrival were 36.6 °C, 59 beats/minute, and 113/56 mmHg, respectively. His laboratory data are shown in Table 1. A urinalysis on arrival showed macrohematuria and proteinuria, and a urinary sediment analysis revealed more than 100 isomorphic red blood cells per high power field. The urinary protein creatinine ratio and albumin creatinine ratio were 7.1 and 4.5 g/g⋅Cr, respectively.

**Table 1:** Laboratory data

Urinary examination		Blood chemistry	
pH (4.5–7.5)	7.5	Glu (mg/dl, 73–109)	80
Protein (g/g⋅Cr)	7.1	TP (g/dl, 6.6–8.1)	6.7
Albumin (g/g⋅Cr)	4.5	Alb (g/dl, 4.1–5.1)	4.6
Occult blood	(3+)	BUN (mg/dl, 8–20)	12.6
Glucose	(−)	Cr (mg/dl, 0.65–1.07)	0.65
β_2_MG (μg/l, < 150)	277	eGFR (ml/min/1.73m^2^)	126.4
NAG (U/g⋅Cr, < 5.6)	5.7	UA (mg/dl, 3.7–7.8)	4.2
RBC (/HPF, < 5)	> 100	Na (mEq/l, 138–145)	141
		K (mEq/l, 3.6–4.8)	4.2
Complete blood count		Cl (mEq/l, 101–108)	106
WBC (/μl, 3300–8600)	4090	Ca (mg/dl, 8.8–10.1)	9.2
RBC (× 10^4^/μl, 435–555)	557	IP (mg/dl, 2.7–4.6)	3.1
Hb (g/dl, 13.7–16.8)	16	AST (U/l, 13–30)	12
Ht (%, 40.7–50.1)	45.4	ALT (U/l, 10–42)	7
Plt (×10^4^/μl, 15.8–34.8)	18	LDH (U/l, 124–222)	127
		ALP (U/l, 38–113)	67
Coagulation		γGTP (U/l, 13–64)	9
APTT (seconds, < 37)	37.2	CRP (mg/dl, 0–0.14)	0.02
PT (seconds, 9.8–12.1)	11.8	IgG (mg/dl, 861–1747)	918
Fib (mg/dl, 200–400)	197	IgA (mg/dl, 93–393)	125
Factor VIII (%, 78–165)	47.6	IgM (mg/dl, 33–183)	76
Factor IX (%, 67–152)	72.4	C3 (mg/dl, 73–138)	71
Factor XI (%, 75–137)	73.8	C4 (mg/dl, 11–31)	20.7
Factor XII (%, 36–152)	61.7	CH50 (U/ml, 31.6–57.6)	50.9
vWF (%, 50–150)	23	CEA (ng/ml, < 5.2)	1.1
vWF antigen (%)	37.3	CA19–9 (U/ml, < 36.8)	8.5
		PSA (ng/ml, < 4)	1.43
Serology			
ANA (< 1:40)	< 1:40		
MPO-ANCA (U/ml, < 3.5)	< 0.5		
PR3-ANCA (U/ml, < 2.0)	< 0.5		
ASLO (IU/ml, < 240)	< 13.6		

*Alb* albumin; *ALT* alanine transaminase; *ANA* antinuclear antibody; *APTT* activated partial thromboplastin time; *ASLO* antistreptolysin-O; *AST* asparate transaminase; *β*_*2*_*MG* β_2_-microglobulin; *BUN* blood urea nitrogen; *C3* complement 3; *C4* complement 4; Ca, calcium; CH50, 50% hemolytic complement activity; *Cl* chloride; *Cr* creatinine; *CRP* C-reactive protein; *eGFR* estimated glomerular filtration rate; *Fib* fibrinogen; *Glu* glucose; *γGTP* γ-glutamyltranspeptidase; *Hb* hemoglobin; *Ht* hematocrit; *IgA* immunoglobulin A; *IgG* immunoglobulin G; *IgM* immunoglobulin M; *IP* inorganic phosphate; *K* kalium; *LDH* lactate dehydrogenase; *MPO-ANCA* myeloperoxidase antineutrophil cytoplasmic antibody; *Na* natrium; *NAG* N-acetyl-β-D-glucosaminidase; *Plt* platelets; *PSA* prostate-specific antigen; *PR3-ANCA* serine proteinase3-anti-neutrophil cytoplasmic antibody; *PT* prothrombin time; *RBC* red blood cells; *TP* total protein; *UA* uric acid; *vWF* von Willebrand factor; *WBC* white blood cells

**Fig. 2:**
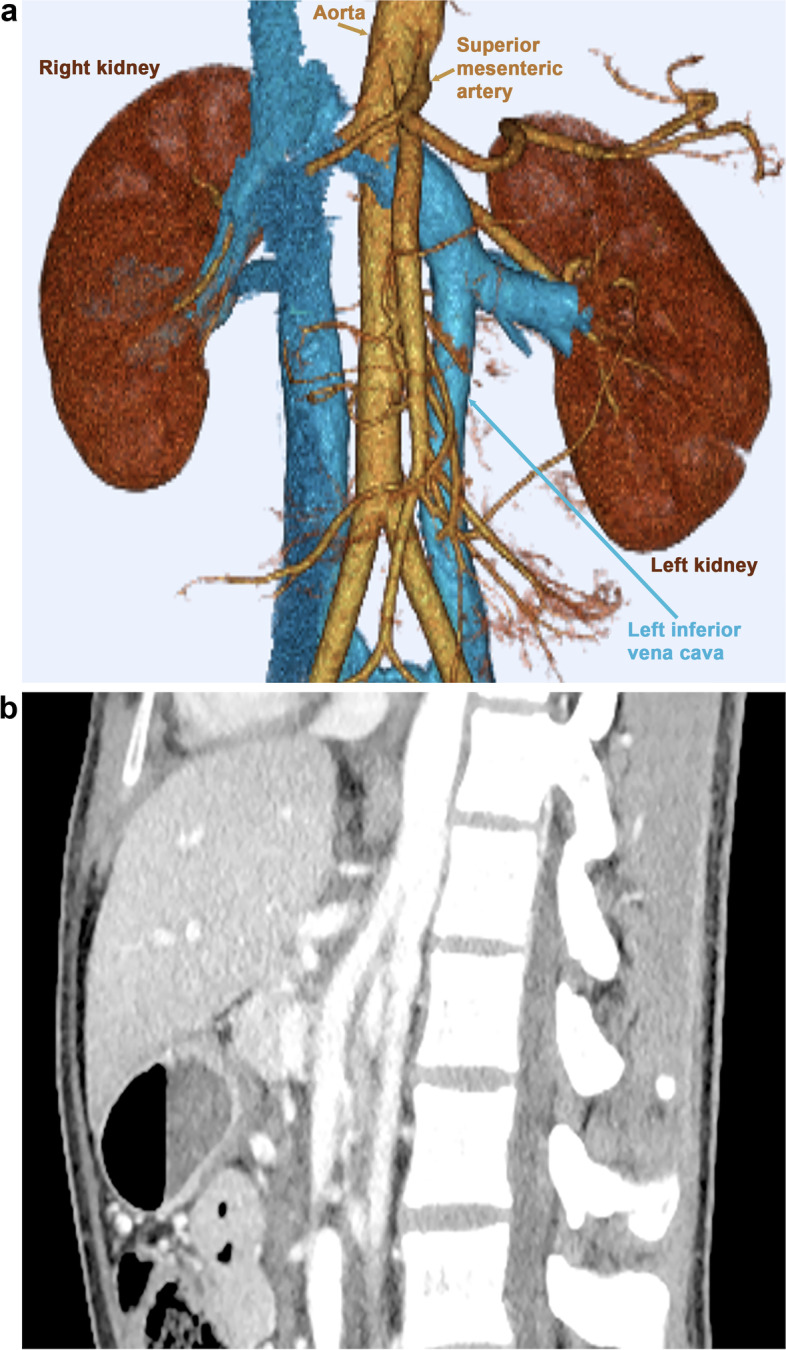
(**a**) A three-dimensional contrast-enhanced computed tomography image. Double inferior vena cava and narrowing (arrowhead) of the left renal vein were observed after interflow of the left inferior vena cava. (**b**) The aortomesenteric angle on sagittal reconstruction of the computed tomography image was 14.7°

**Fig. 3:**
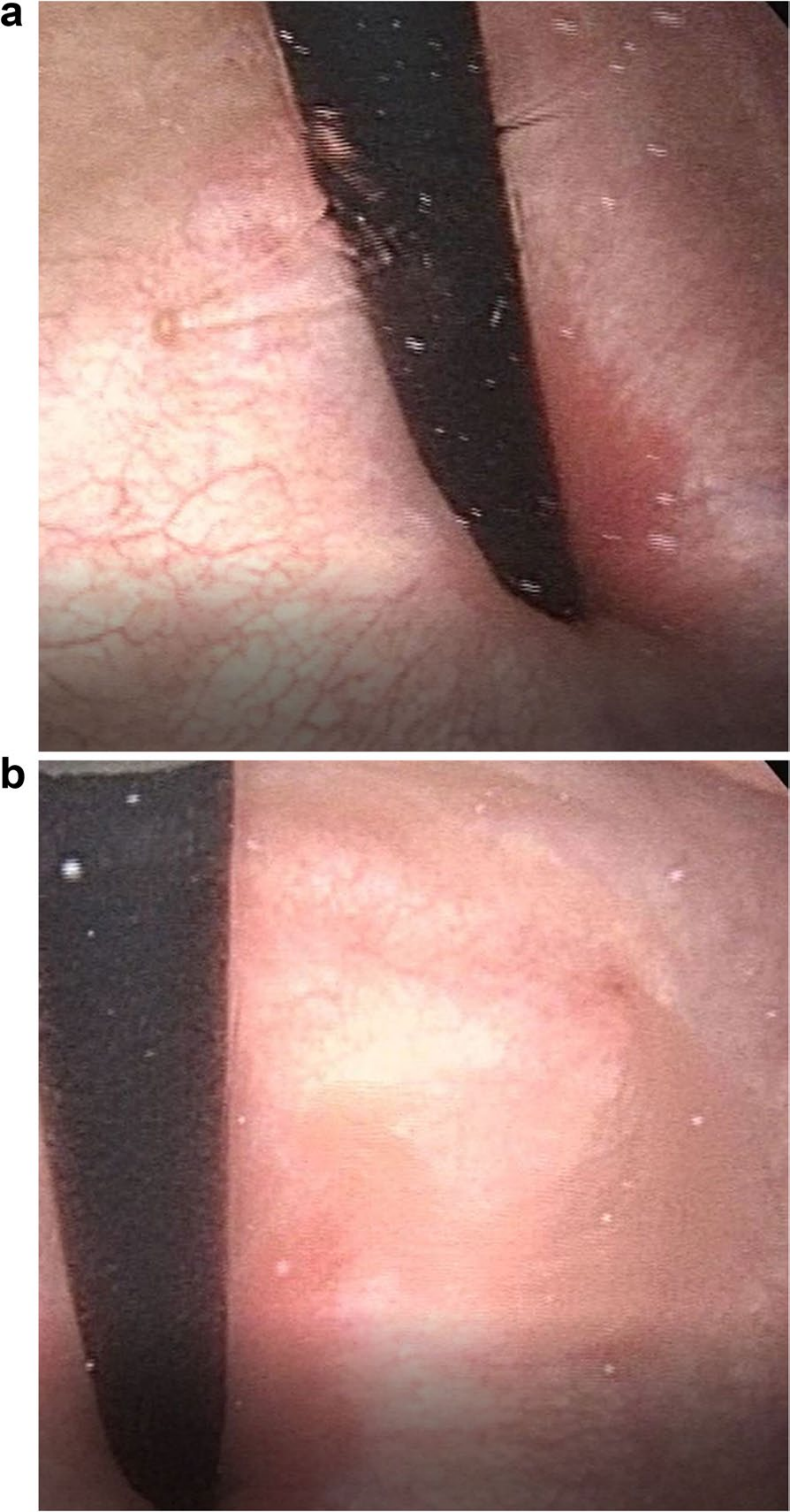
(**a**) The flow of normal urine from the right ureteral opening. (**b**) The flow of macrohematuria from the left ureteral opening

Three-dimensional contrast-enhanced CT showed double IVC and narrowing of the LRV after interflow of the left IVC (Fig. 2a, Supplementary Video S1). The aortomesenteric angle on sagittal reconstruction of the CT image was 14.7° (Fig. 2b). Cystoscopy revealed a flow of macrohematuria from the left ureteral opening (Fig. 3a, b, Supplementary Video S2, and S3). Under the suspicion of NCS, Doppler ultrasonography was performed. The ratio of the LRV diameter at the hilar/aortomesenteric portion was 2.9 (6.7/2.3 mm) and the peak velocity at the aortomesenteric portion was 29 cm/s with a pressure gradient of 0.33 mmHg; ultrasonography provided little evidence to raise the suspicion of the nutcracker phenomenon. On physical examination, no left varicoceles were apparent.

**Fig. 4:**
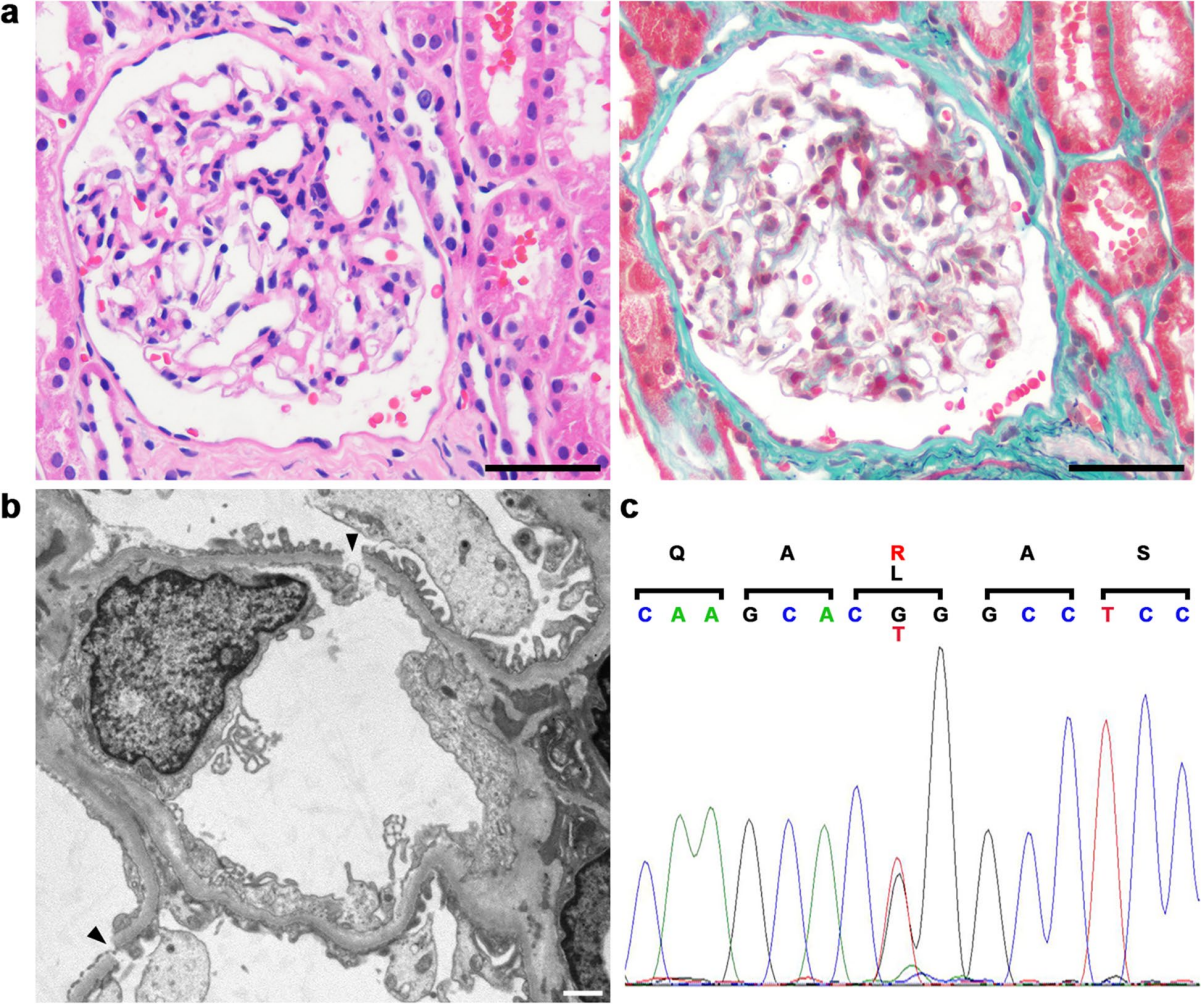
(**a**) Light microscopy showed red blood cells in Bowman’s space and the tubular lumen with hematoxylin and eosin staining (left panel) and Masson trichrome staining (right panel). Bars = 50 μm. (**b**) Transmission electron microscopy showed glomerular basement membrane disruption (arrowheads). Bar = 1 μm. (**c**) A genetic analysis revealed a heterozygous variant at c.4793 T > G (p.L1598R) in the *COL4A3* gene

Since the severe albuminuria (3.2 g/g⋅Cr) continued at 5 days after the initial presentation, he was admitted to our hospital to receive a left kidney biopsy. There were 18 glomeruli, which showed minor glomerular abnormalities. Light microscopy showed red blood cells in Bowman’s space and the tubular lumen (Fig. 4a). An immunofluorescence study was negative for immunoglobulin G (IgG), IgA, IgM, complement 3 (C3), C1q, and fibrinogen. Electron microscopy revealed disruptions of the glomerular basement membrane (GBM) (Fig. 4b, Supplementary Fig. 1). He was discharged without any complications after kidney biopsy and was followed up as an outpatient. Doppler ultrasonography was repeated; however, the parameters did not show a major difference between the supine and upright position. His macrohematuria and proteinuria improved gradually without treatment. Although he experienced intermittent macrohematuria at home after discharge four times, neither proteinuria nor microhematuria were observed during follow-up examinations in the outpatient clinic at one, two, five, eight and 14 months after discharge.

As vulnerability of the GBM was suspected based on electron microscopy, genetic analyses of *COL4A3* and *COL4A4* were performed, which revealed a heterozygous variant at c.4793 T > G (p.L1598R) in the *COL4A3* gene (Fig. 4c). Screening for coagulation disorders revealed that the patient’s factor VIII and von Willebrand factor (vWF) values were low at 47.6 and 23%, respectively. A multimer analysis of vWF showed a normal multimer pattern and he was diagnosed with von Willebrand disease type 1. As the bleeding tendency was mild, replacement of factor VIII was not performed.

## Discussion and conclusions

We experienced a case with macrohematuria and proteinuria. Although repeated Doppler ultrasonography did not show evidence of NCS, the aortomesenteric angle on sagittal reconstruction of the CT image was 14.7°, which fulfilled the criterion of < 35° for the diagnosis of NCS [1]. The present case also had double IVC, which might have predisposed the patient to NCS, as was described in a previous report [9]. As macrohematuria in NCS was explained by rupture of the thin-walled varices due to LRV hypertension [12], severe albuminuria was thought to be unusual in the present case and the left kidney biopsy was performed. Light microscopy confirmed the presence of red blood cells in both Bowman’s space and the tubular lumen and disruption of the GBM was confirmed by electron microscopy, suggesting that the macrohematuria and proteinuria originated from glomerular hemorrhage. The isomorphic hematuria in the present case might represent the severity of the disruption of the GBM, since glomerular hemorrhage usually appears as dysmorphic hematuria. A previous case in a single kidney showed nephrotic range proteinuria due to NCS; however, a kidney biopsy was not performed, and the origin of the proteinuria was unknown [13].

**Fig. 5:**
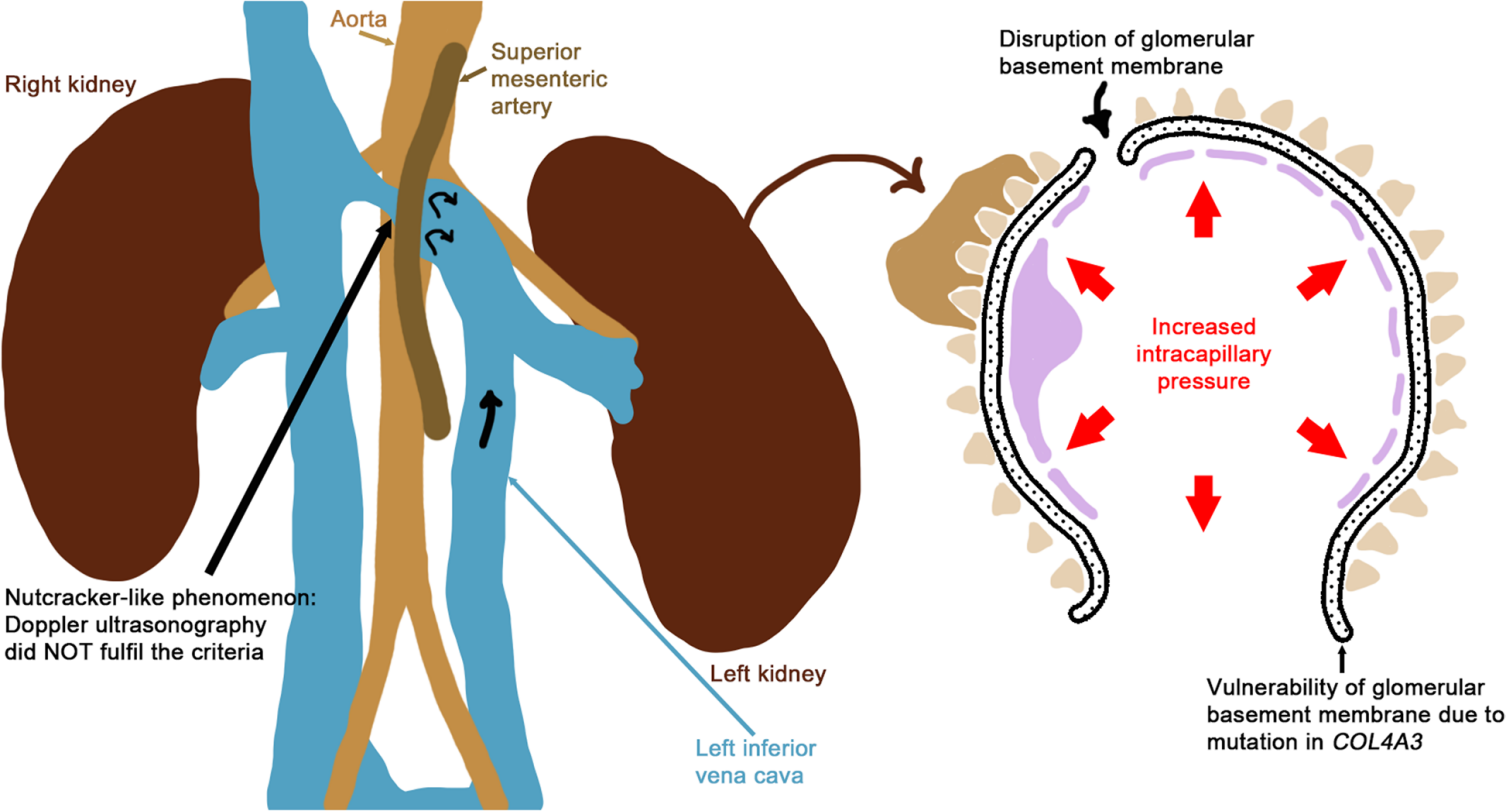
A hypothetical mechanism

In the present case, a genetic analysis was performed to investigate the origin of the vulnerability of the GBM and a heterozygous variant was identified at c.4793 T > G (p.L1598R) in the *COL4A3* gene. A previous report showed that a patient with c.4793 T > G in *COL4A3* and c.448G > C in *COL4A5* had irregular thinning of the GBM while his father with c.4793 T > G in *COL4A3* did not show hematuria [14]. Another previous report analyzed 24 patients with autosomal recessive Alport syndrome, 17 of which had mutations in *COL4A3* [15]. Of the 17 patients, 13 had compound heterozygous mutations in *COL4A3*, 5 of which had c.4793 T > G in *COL4A3*. Among the parents of the 5 patients, only one mother with c.4793 T > G in *COL4A3* had hematuria; the other parents seemed normal [14]. As the thinness of the GBM in the present case was not obvious and hematuria had not been pointed out previously, c.4793 T > G in *COL4A3* might not have affected the synthesis of the triple helical molecule of the type IV collagen α3/4/5 chains. However, the disruption of the GBM in the present case implied that p.L1598R in the carboxy-terminal non-collagenous 1 (NC1) domain of the type IV collagen α3 chain may affect the associative strength between the type IV collagen triple helical molecules. The spontaneous remission of proteinuria in the present case might imply that the disruption of the GBM was repaired by newly synthesized collagen molecules after the end of the long drive. The hypothetical mechanism of the present case is shown in Fig. 5.

The patient in the present case had been diagnosed with NS due to *RIT1* mutation. While there is a case report of a patient with nephrotic syndrome due to crossed fused ectopic kidneys in NS [16], nephrotic range proteinuria in NS is rare. Coagulation defects were reported in 31% (4/13) of NS patients with *RIT1* mutations [17]. The present case had low factor VIII and vWF activity with a normal multimer pattern, which was compatible with von Willebrand disease type 1. Although the coagulation disorders in the present case might have partially affected the onset of NCS, macrohematuria spontaneously improved in the present case and his von Willebrand disease type 1 was thought to be mild since the vWF antigen value was 37.3%.

In conclusion, the present case showed macrohematuria and proteinuria due to NCS in NS with double IVC and von Willebrand disease type 1, and the origin of the macrohematuria and proteinuria came from glomerular hemorrhage because of vulnerability of the GBM due to *COL4A3* mutation.

## Supplementary Information

**Additional file 1: Supplementary Video S1.** A 3D reconstruction of the entire left IVC subsystem.

**Additional file 2: Supplementary Video S2.** The flow of normal urine from the right ureteral opening.

**Additional file 3: Supplementary Video S3.** The flow of macrohematuria from the left ureteral opening.

**Additional file 4: Supplementary Fig. S1.** Transmission electron microscopy

## Data Availability

The datasets used and/or analyzed during the current study are available from the corresponding author on reasonable request.

## Abbreviations

C3: Complement 3
CT: Computed tomography
GBM: Glomerular basement membrane
IgG: Immunoglobulin G
IVC: Inferior vena cava
LRV: Left renal vein
MAPK: Mitogen-activated protein kinase
NC1: Non-collagenous 1
NCS: Nutcracker syndrome
NS: Noonan syndrome
vWF: Von Willebrand factor
