# Supplementary figures and images for: Disruption of the glomerular basement membrane associated with nutcracker syndrome and double inferior vena cava in Noonan syndrome: a case report
Source: BMC Nephrol. 2022 Feb 12;23:65. doi: 10.1186/s12882-022-02671-4 (PMC8841073; doi:10.1186/s12882-022-02671-4)

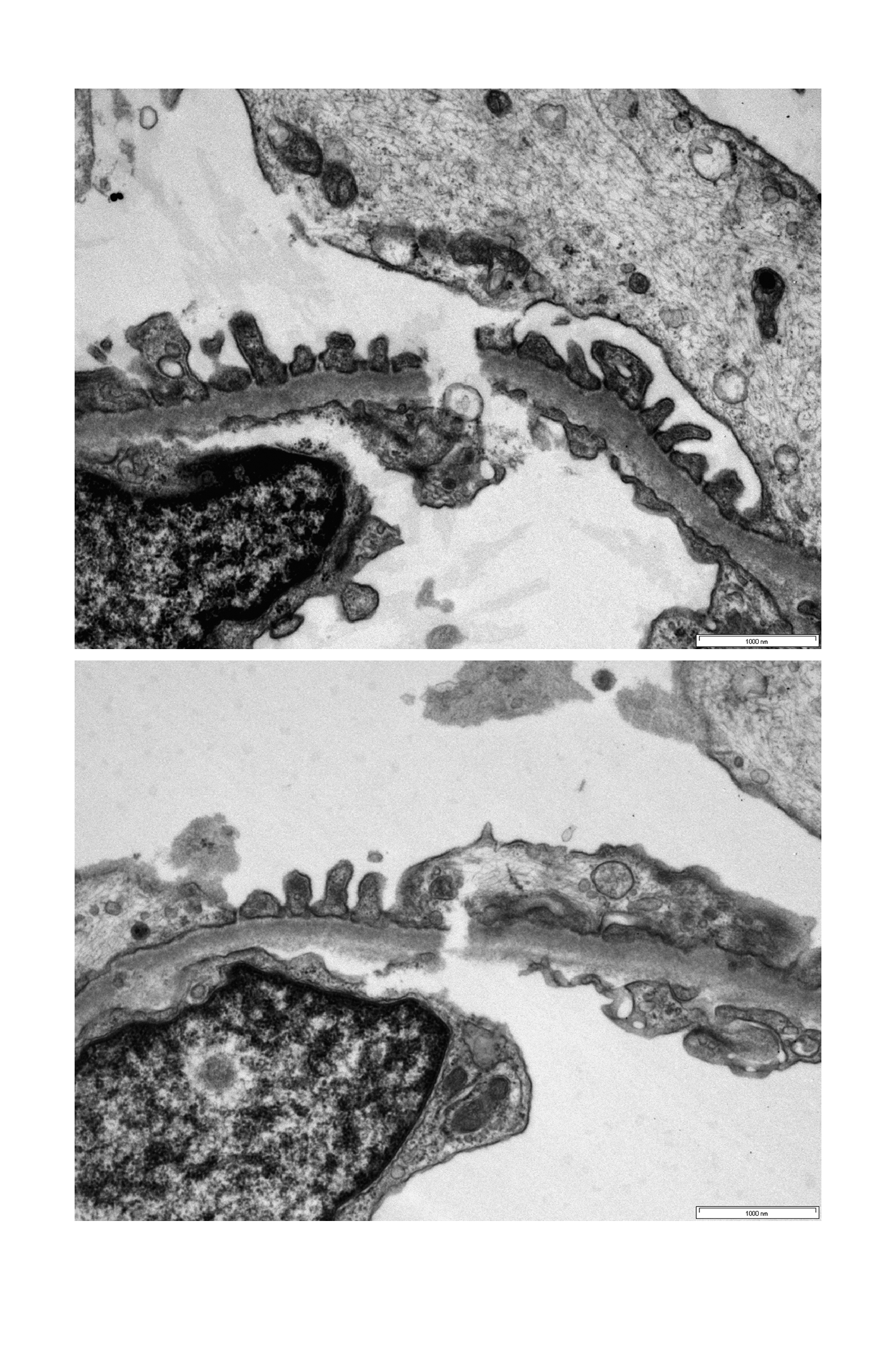

Supplement: Supplementary file 4 — Additional file 4: Supplementary Fig. S1. Transmission electron microscopy [file 12882_2022_2671_MOESM4_ESM.tif]
